# Infra-specific folk taxonomy in sorghum (*Sorghum bicolor *(L.) Moench) in Ethiopia: folk nomenclature, classification, and criteria

**DOI:** 10.1186/1746-4269-3-38

**Published:** 2007-12-27

**Authors:** Firew Mekbib

**Affiliations:** 1 Haramaya University, PO Box 138, Dire Dawa, Ethiopia; 2 Norwegian University of Life Sciences, Department of Plant and Environmental Sciences, PO Box 5503, N-1432, Aas, Norway; 3 PO Box 485 code 1250, Addis Ababa, Ethiopia

## Abstract

**Background:**

Sorghum is one of the main staple food crops for the poorest and most food insecure people of the world. As Ethiopia is the centre of origin and diversity for sorghum, the crop has been cultivated for many thousands of years. Hence, indigenous knowledge based sorghum classification and naming has a long tradition.

**Methods:**

In order to assess folk taxonomy, various research methods were employed, including, focus group interviews with 360 farmers, direct on-farm participatory monitoring with 120 farmers, key informant interviews with 60 farmers and development agents and semi-structured interviews with 250 farmers. In addition, diversity fairs were conducted with over 1200 farmers. Assessment of folk taxonomy consistency was assessed by 30 farmers' evaluation of 44 folk species.

**Results:**

Farmers have been growing sorghum for at least 500 years (20 generations). Sorghum is named as *Mishinga *in the region. Farmers used twenty five morphological, sixty biotic and abiotic and twelve use-related traits in folk taxonomy of sorghum. Farmers classified their gene-pool by hierarchical classifications into parts that represented distinguishable groups of accessions. Folk taxonomy trees were generated in the highland, intermediate and lowland sorghum ecologies. Over 78 folk species have been identified. The folk species were named after morphological, use-related and breeding methodology used. Relative distribution of folk species over the region, folk taxonomy consistency, and comparison of folk and formal taxonomy are described.

**Conclusion:**

New folk taxonomy descriptors have been identified and suggested to be used as formal taxonomy descriptors. It is concluded that integrated folk-formal taxonomy has to be used for enhanced collection, characterisation and utilization of on farm genetic resources.

## Background

Sorghum is one of the main staples for the world's poorest and most food insecure people. The crop is genetically suited to hot and dry agro-ecologies where it is difficult to grow other food crops. Developing countries account for roughly 90% of the world's sorghum area and 77% of the total output [1]. In developing countries, the lion's share of the crop is grown by small scale farming households operating at the margins of subsistence.

Sorghum is the most important staple crop in Ethiopia. It is grown on 1,468,070 ha with a total production of 2,173,598 Mt [2]. It accounts 14.2% and 13.6% of the crop area and production respectively. Ethiopia is the centre of origin and diversity for sorghum [3], the crop has been cultivated for many thousands of years and hence indigenous knowledge based sorghum classification and naming has a long tradition.

Sorghum was first described by Linnaeus in 1753 under the name of *Holcus*. Moench later separated the genus sorghum from the *Holcus *and made the combination of *Sorghum bicolor*. The current formal taxonomic concept of the sorghum genus and species agrees with the one established by Moench. All the different names given by the various taxonomists and are hence taken as synonym to *S. bicolor *(L.) Moench. The classification of Sorghum genus was attempted by Brotero (1804), Roxburghii (1820), Steudel (1854), Chiovenda (1912), Piper (1915), Stapf (1917) (as cited in [4]). The most detailed classification was made by Snowden [5,6]. He described 31 cultivated species and 17 related wild species and gave 48 different types that are well defined by a number of distinct characters. After decades of bio-systematic research, Harlan and de Wet [7] have developed a simplified classification useful to plant scientists. The cultivated taxa, covering 28 (out of 31) species of Snowden's series in de Wet [8], are partitioned into the five basic races and ten hybrid races under *Sorghum bicolor *subsp. *bicolor*.

Taxonomy, as described by Harlan [9] is pragmatically the science of convenience that normally reveals genetic affinity and evolutionary history. However, the inconsistencies and lack of agreements among taxonomists working even on the same genus and species is remarkable [9]. In view of these discrepancies among formal taxonomists, most plant scientists have developed their own informal and intuitive classifications based on experience, as to what constitute useful groupings. Similarly, farmers, who have been domesticating, developing and cultivating crops for hundreds of years, use their own classification system called informal, farmer or folk taxonomy. The rationale behind folk taxonomy has been studied very recently in taro (*Colocasia esculenta *Schott) [10], cassava (*Manihot esculenta *Crantz) [11] and rice [12].

In order to utilize and maintain on farm genetic diversity, farmers have been classifying, naming and grouping their varieties for millennia using different folk taxonomy descriptors. Folk nomenclature and taxonomies not only create labels and keys to distinguish morphological differences [13] but they are indicators of other non-morphological varietal attributes also.

Related study made on farmers' breeding of sorghum [14,15] and the farmers' seed systems [16,17] have indicated that farmer's varieties have been developed, identified, diffused and maintained on farm for a long period. How are these varieties named? Who name them? How is the name used in the course of diffusion? Is there any pattern of classification? Are there descriptors for folk taxonomy of sorghum? The few attempts made in Ethiopia, in North Shewa and South Wello [18] focused only in few formal taxonomic descriptors; and Western Hararghe [19] cited the presence of folk taxonomic classification only. These studies have not treated folk taxonomy from holistic perspectives, i.e., from the general knowledge base that determines folk taxonomy: naming, classification, and criteria; identification of folk species and consistency in the indigenous technical knowledge.

Hence the objectives of this study were:

1. To characterise folk taxonomy: classification, naming, criteria

2. To identify the folk species, subspecies and varieties in the region

3. To assess the consistency in folk taxonomy and compare it with formal taxonomy

## Methods

### Study areas

**Figure 1:**
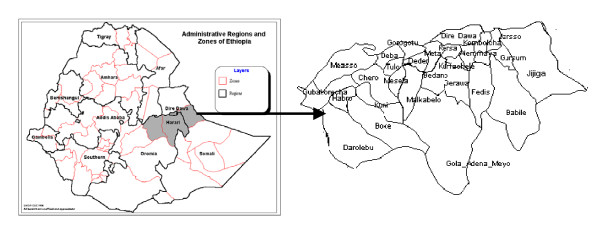
Map and Position of the study region in Ethiopia. Detail *wereda *map of the study region.

Eastern Ethiopia (Figure 1) was selected for the following reasons: first, the crop has coexisted with the people for millennium and sorghum production is predominantly based on farmers' varieties [14]. Hence, it is suitable for studying farmers' indigenous classification and nomenclature system; second, the region is rich in farmers' varieties and hence suitable to study folk taxonomy; third, folk species are distributed all over the region, which helps to assess the consistency in folk taxonomy; fourth, as folk species and varieties are there, then it is easier to develop folk taxonomy tree.

### Survey research methodologies used

Diverse research methodologies have been used in order to understand the whole scenario of sorghum folk taxonomy.

#### Focus group interviews

First, community-based Participatory Rural Appraisals were made in 12 farmers associations of highland, intermediate and lowland areas and then participants were seconded by the community based on wealth and gender, to know how they classify and name sorghum. Over 360 farmers in groups were interviewed. With regard to gender, either male or female, only one member of the household is included in the group. The selected weredas (districts) for this study were from the highland: Girawa and Hirna; Intermediate: Alemaya and Hirna and from Lowland: Babile and Dire Dawa.

#### Direct participatory on farm monitoring

One hundred twenty farmers were directly monitored on farm, over the selected weredas in the three sorghum crop ecologies, for assessing folk taxonomy.

#### Key informant interviews

To assess the general folk taxonomy of sorghum key informants, up to five per farmers associations, Ministry of Agriculture crop production experts, non-governmental organizations in each site, were interviewed. Most of the findings were discussed; on workshops (Plate 1) held with farmers and other key informants.

#### Semi-structured interviews

These interviews were carried out with 250 farmers to quantify the folk taxonomy all over the region.

#### Diversity fairs

These were one of the tools employed for assessing the knowledge and folk species of the farmers. These were held around the time of physiological maturity of the crop. An average of 50 farmers each participated in the 24 diversity fairs, making a total of 1200 farmers. Both women and men brought all the varieties grown in their field to the fair and discussed every folk species/varieties name and character (Plate 2, 3, 4). The varieties brought by the farmers were piled and sorted into different folk species, varieties, and sub varieties level.

### Field experiments

Folk species/varieties were planted in Babile, Haramaya University Research Substation, in 3 m lengths of two rows plot for checking of consistency in folk taxonomy. The dominant 44 folk species grown in the region were named and identified by 30 farmers to check consistency in folk taxonomy.

### Data analysis

Collected survey and field experiment data were subjected to descriptive statistics, log linear regression, and cluster analysis using SPSS Ver. 10 and STATISTICA statistical softwares.

## Results

### Naming of sorghum as 'Mishinga'

Farmers laughed when I asked where sorghum comes from and who named it as *Mishinga*. Farmers said that what type of question is this. Our children have never asked us. Discussion continued and finally consensus was reached that sorghum has to come from somewhere and named by someone. *Bishinga *or *Mishinga *(semantic names) is the local name given for sorghum in *Oromiffa*, one of the Ethiopian languages. They said that this name was given by Adem for sorghum. *Mishinga *means edible; it means '*Naema*' in Arabic. It is called *Mashilla *by *Amhara *farmers. It has been here for 20 generations; i.e., for almost over 500 years. Actually, farmers indicated that, as far as their knowledge goes, the first human food was sorghum.

### General folk classification scheme

In understanding folk taxonomies, the following steps were pursued 1) recording the folk species grown in the zones, *weredas*, and farmer associations 2) sorting the identified species using farmers' descriptors and 3) develop the folk taxonomy tree. This was done with the group of farmers, key informants and individual farmers in each study site.

**Figure 2:**
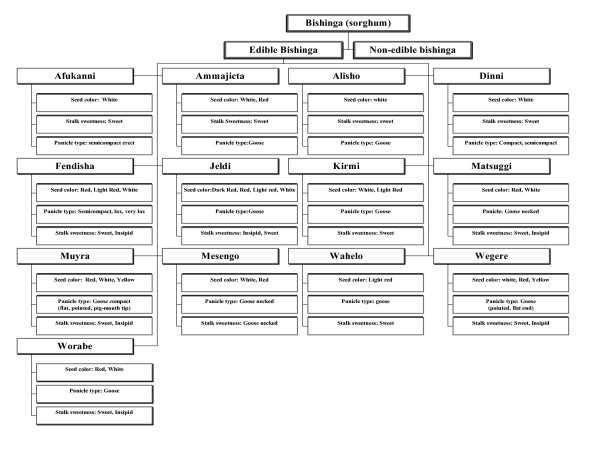
Folk taxonomy tree in the lowland ecology.

**Figure 3:**
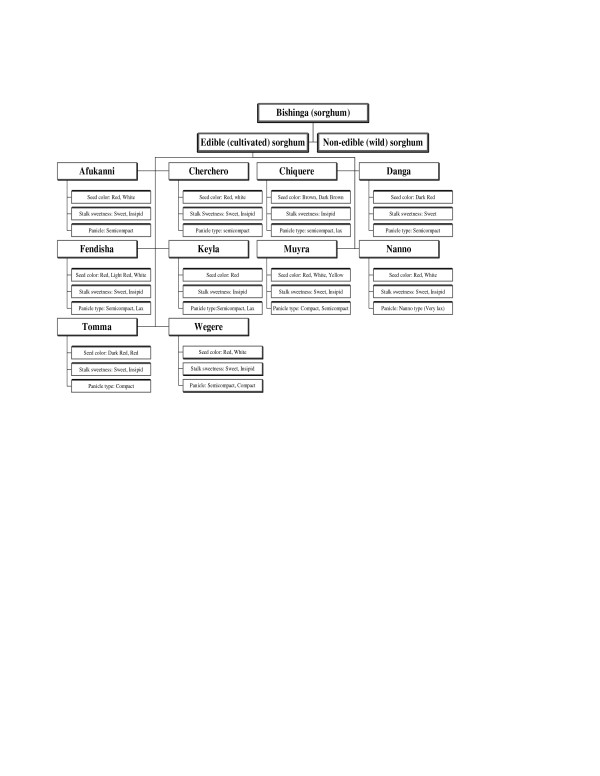
Folk taxonomy tree in the intermediate ecology.

Folk sorghum taxonomy is farmers' classifications, naming, and grouping of sorghum. Folk species are farmers' taxonomic unit of classification. A folk species has folk varieties, and a folk variety has subvarieties. In folk taxonomy, farmers used descriptors that are grouped into four types. These are botanical, use, agro-ecological, and technological-related descriptors. The class of folk taxonomy descriptors and examples of each are indicated (Table 1). The relative importance of folk taxonomic criteria is shown (Table 2). The way the descriptors are used for folk taxonomy is shown in Table 3, 4, 5, 6, 7, 8 and Figure 2, 3, 4. The folk taxonomy is consistent at all four spatial levels viz., generic, species, variety, and sub-variety levels, when judged by the various farmers' folk taxonomic criteria. Sorghum folk names are, thus, meaningful both within and beyond farmers associations and *wereda *boundaries. Folk species varied in morphological characters that are commonly employed as phenotypic markers for folk taxonomy.

**Table 1:** The general folk classification criteria and some of the farmers' descriptors, descriptors class and examples

**Class of characters**	**Descriptors**	**Some descriptors class**	*Example*
Botanical	Panicle type	Very compact, goose flat end	*Muyra, Jeldi*
		Very compact, goose with pointed end	*Muyra, Jeldi*
		Very compact, goose with pig-mouth end	*Muyra, Wegere*
		Semicompact, goose flat end	*Fendisha*
		Semicompact, goose oval	*Wegere, Ammajicta*
		Semicompact, erect oval	*Kuaffa Kassa*
		Very loose with erect rachis and dropping branches to one direction	*Fechatee, Nanno, Fendisha*
		Very loose with no rachis and dropping branches to all direction	*Fechatee, Nanno*
	Seed colour	Reddish brown	*Fitibile*
		White	*Arebe*
		Straw (ashy)	*Wegere*
		Chalky white	*Wegere*
		Grey	*Muyra*
		Purple	*Chamme, Worabe*
		Red	*Muyra*
		Light red	*Muyra, Fendisha*
		Light brown	*Danga*
		Brown	*Fitibile*
		Yellow	*Wegere*
		White and Red	*Tufkuur*
	Plant height	Tall	*Fendisha*
		Medium	*Wegere*
		Short	*Gebabe*
		Very short	*Jilbeb*
	Awns	Strong awn	*Kirmi*
		Weak awn	*Fendisha*
		Awnless	*Muyra*
Use	*Injera*	Excellent	*Fendisha*
		Very good	*Nanno*
		Good	*Wegere*
		Poor	*Muyra*
Agro-ecological	Drought	Resistant	*Ammajicta*
		Moderate	*Muyra*
		Susceptible	*Fendisha*
	Cold tolerance	Resistant	*Merturasse, Fechatee*
		Moderate	*Gebabe*
		Susceptible	*Wegere, Arebe*
Technological	Lodging	Resistant	*Ammajicta, Jilbeb*
		Moderate	*Gebabe*
		Susceptible	*Wegere, Muyra*
	Bird resistance	Resistant	*Kirmi, Firekolef, Fitibile*
		Moderate	*Gebabe*
		Susceptible	*Wegere, Muyra*

**Table 2:** Classification criteria: Percent of farmers who are using various traits for folk taxonomy

**Morphological Traits**	H (N = 101)	I (N = 99)	L (N = 50)	Total (N = 250)
Seedling vigor (NS)	94	86.8	82	88.8
Leaf number (*, §	92	89.9	74	87.6
Plant colour (NS)	93	89.9	92	91.6
Leaf midrib colour(NS)	96	98	98	97.2
Plant height (NS)	99	96.9	100	98.4
Nodal tillers (NS)	96	87	94	92.4
Basal tillers (NS)	98	90	94	94
Flower synchrony for tillers (NS)	92	87	94	90.8
Internode length (NS)	93	91	88	91.2
Node number (NS)	93	91	80	87.2
Awns(*, §	96	87.8	82	90
Panicle type (C)	100	100	100	100
Panicle size (NS)	100	97.9	100	99.2
Glume colour (*, §	97	89	100	95.2
Grain coverage (NS)	99	94.9	100	97.6
Grain size (NS)	100	98.9	100	99.6
Grain/seed price (NS)	97	97.9	96	97.2
Seed colour (c)	100	100	100	100
Grain plumpness (fill) (NS)	99	98	94	97.6
Threshability (c)	99	100	100	100
Seed shattering (*, §	84	91	70	84
Leaf greenness (less senescence) (NS)	95	93	84	92
Stalk sweetness (c)	99	100	100	100
Maturity (*, §	99	95	100	98
Plant height (c)	100	100	100	100

**Biotic and abiotic stress related traits**				

Reaction to weevil (NS)	30.6	39	50	38
Stalk borer resistance (NS)	31.7	31.3	48	34.8
Shootfly resistance (*, §	24	17.2	36	23.6
Aphid (*Kishkish*) (NS)	10.9	13	18	13.2
Leaf disease resistance(*, §	25.7	14	32	22.4
Resistance to storage fungi (NS)	15.8	10	44	19.2
Grain mold (NS)	15.8	10	18	14
Pokkah boeng (*Harkan*) (*, §	17.8	8	30	16.4
Head midge (*, §	13.9	9	32	15.6
Ergot (NS)	15.8	15.1	12	14.8
Smut resistance (NS)	21.8	17	28	21.2
Lodging resistance (*, §	84	64.6	86	76.8
Drought resistance (*, §	78	87.8	96	85.6
Frost tolerance (NS)	16.8	8	10	12
Low soil fertility tolerance (NS)	90	88.9	90	89.6
Water logging tolerance NS)	20.8	17.2	20	19.2

**Use related traits**				

Stalk marketability (NS)	44	57.6	46	49.6
Yield (C)	100	100	100	100
Biomass production (Leaf +stalk) (NS)	99	100	100	99.6
Taste (NS)	100	98.9	96	98.8
Milling quality(NS)	23.8	25.2	36	26.8
Boiled grain (*Shumo*) (NS)	99	100	100	99.6
Porridge (*Lafiso*) (NS)	99	98	98	99.2
Quality and storability of 'injera' (C)	100	100	100	100
Flour-to-water ratio NS)	100	98.9	98	99.2
Fuel wood value (*, §	96	98	82	94
Animal feed value (c)	100	100	100	100
Construction value (NS)	79	68	76	74

Legend: H = Highland, I = Intermediate, L = Lowland; * and §indicates significance of chi-square (X^2^) and likelihood ratio (G^2^) respectively at 5%; NS = non significant at 5%;C = Similar value hence no computation of X^2 ^and G^2^

**Table 3:** A simple stepwise classification from generic to sub-variety level

Folk generic level	Folk sub-generic level (optional)	Folk species level	Folk variety	Folk subvariety level
Bishinga	Cultivated1 (edible) sorghum	Muyra	*Muyra Red*	*Muyra red long sweet stalked*
			*Muyra Brown*	
			*Muyra Yellow*	
			*Muyra Dark Red*	
	Wild^2 ^(non-edible) sorghum	*Degengof*		
		*Keelo (Harchatee)*		
		*Firekolef*		

Legend:^1^**edible sorghum**-sorghum used for food, feed, fuel wood and construction and are cultivated, selected and maintained by the farmers

^2^**non-edible sorghum**-sorghum with no common uses and are not grown, selected and maintained by the farmers but are self-grown or -propagated on the farm without farmers knowledge or selection

**Table 4:** Farmers' criteria for separating wild and cultivated types

Characters for comparison	Wild (non-edible) types **(e.g. *Harchatee*)**	Cultivated (edible) types **(e.g. *Muyra*)**
Panicle type	Lax	Compact to semi-compact
Glume colour and coverage	Full	Less
Shattering level	High	Low sometimes absent
Tillering	Very high	Few to single stem
Stem thickness	Thin	Thick
Seed size	Very small to small	Small to big

**Table 5:** The possible number of varieties that can be generated from a folk species by using various characters

Sorghum folk species	Colour	Panicle type	Height	Stalk sweetness
*Fendisha*	White	Compact	Long	Sweet
	Light Brown	Semi-compact	Medium	Insipid
	Brown	Lax	Short	
	Grey			
*Wegere*	White	Semi-compact	Long	Sweet
	Brown	Compact	Medium	Insipid
	Yellow			
*Muyra*	Light Brown	Compact flat end	Long	Sweet
	White	Compact pointed-end	Medium	Insipid
	Grey	Compact pig mouth end	Short	
	Brown	Compact with four titts		

**Table 6:** Folk species named after the introducer or name or place of the origin

**Variety**	**Named after**
*Alisho*	Farmer who brought it from Assebot area
*Arebe or Yemeni*	Yemen, name of an Arabic country
*Bisidimo*	Bisidimo, name of a place
*Cherchero*	Chercher, name of a place
*Dinni*	Dinni, name of a farmers association
*Dulla*	Farmer Dulla who introduced to the area
*Engidawork*	Farmer 'Engidawork' and it is introduced from North Shoa
*Kassim*	Introducer Kassim
*Manahaile*	Introducer Manahile
*Mullu*	Mullu, name of FA
*Mureta*	Mureta, name of a place
*Wobere*	Wobera, name of a place
*Wahelo*	Wahelo, name of a place
*HajiiAli*	Introducer '*Hajiiali*'
*Jeldi*	A sorghum that comes with the cover of holy Koran book
*Weliso*	Weliso, name of a place

**Table 7:** Folk species after use and use related traits

**Variety**	**Named after**
*Alasherif*	'*Sheremute*' only for sweet stalk
*Chomme*	'*Chomma*' 'Seed with a lot of fat'
*Daddu*	Sweet as butter
*Fendisha*	Means very beautiful.
*Harkebasse*	Available in times of problem
*Hado*	Soury not good for green eating (*Eshet*)
*Bare Bekeyes*	'*Gan seber*' produce a lot of dough (*Siboka yiberketal*), low flour-to-water ratio
*Kuna*	Sweet stalk, eaten as '*Eshet*', green

**Table 8:** Folk species named after morphological traits

**Varieties**	**Morphological attributes associated with the naming**
*Aday*	White seeded
*Afukanni*	A variety that always gives yield or that never fails to give yield
*Alemo(Ala-mo)*	That quenches thirsty
*(H)Anchero*	Lax type panicle
*Bele Melik*	Drought escaper, early maturing
*Bullo*	Light white and grey colored
*Butene*	Short and thin
*Chamme*	Grows fast
*Chelle*	Amber colored seed
*Chiquere*	Dark red-named after a bird called *Chiquele*, the seed is dark red and leaf is deep green.
*Daslee*	Strong and adaptive
*Fechee*	Lax type of panicle
*Firelemi*	Twin seeded
*Firekolef*	Glume encloses the seed completely
*(H)Ifaato*	Boring for harvesting and threshing because it gives high yield
*Gebabe*	Short statured
*Jilbeb*	'*Tegneto yemiweled' *(*Gabaabdu hortuu*) very dwarf and yielding, very short
*Keyla*	Red seeded
*Kirmi*	Awned panicle, bird resistant
*Kuffa Kassa*	That strengthen the weak
*Kumash*	Early maturing bullo
*Masengo (Kelafo)*	A sorghum type used for beef fattening or come from Kelafo area
*Matsugi*	White seed colour
*Meleta*	Early maturing, drought escaping variety
*Merturasse*	*Mertu *'ladies name'; *Rasse *means loose hair. Dispersed panicle, very lax panicle
*Nanno*	Similar with traditional women hair dressing '*shurube*-traditional tightly plaiting of hair'
*Solle*	Beautiful, named after the seed colour 'very shiny and attractive colour'
*Sheridon*	Early maturing variety
*Tomma*	Broom type of panicle
*Wegere*	It means '*we agre'*-we saw a new variety
*Worabe*	The panicle shape looks like hairs of hayena
*Wourdi*	A variety that grows fast-early maturing
*Tufkuur or Bure*	Mixed colored or miracle of God or surprising sorghum or unique sorghum. Variegated type
*Yade*	Missed (love) its sweet stalk
*Marur*	Shattering
*Umrhajeria*	A seed colour is like whitish stone/rock
*Ilimijamma*	Erect panicle

**Figure 4:**
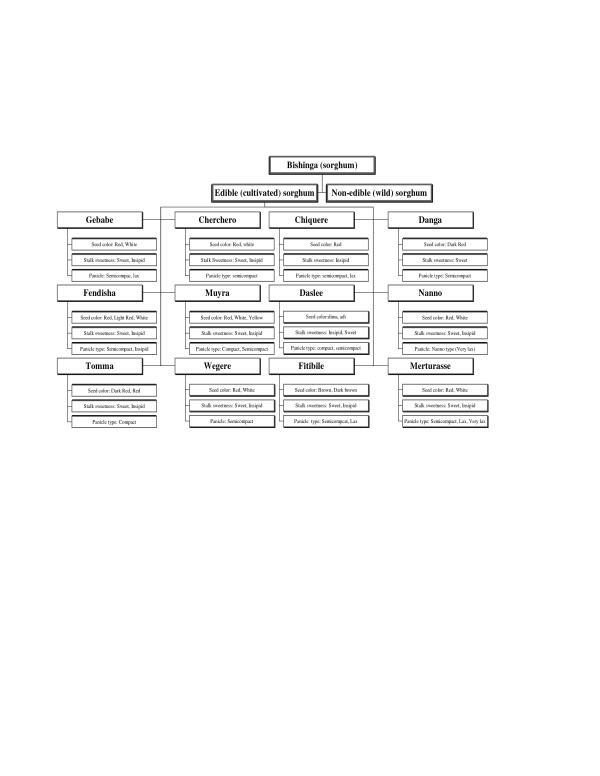
Folk taxonomy tree in the highland ecology.

They varied for seed colour, yield, panicle type, biotic and abiotic stresses resistance, maturity time, feed and food value (Plate 5, 6).

### Folk classification criteria or characters used for folk taxonomy: frequency of usage

The folk taxonomy is based on folk descriptors for classifying the existing genetic diversity. Initially the folk descriptors were suggested in *Oromiffa *language and later they were translated into closer IBPGR/ICRISAT descriptors. The frequency of usage criteria (Table 3) is highly dictated by the level of prevalent total genetic diversity or diversity for the specific traits present on farm. These characters can be grouped into morphological, biotic stress, abiotic stress, and use-related traits. These classes of folk descriptors are used to discriminate one from another of folk species, varieties and sub-varieties.

#### Morphological related traits

these traits span from seedling to maturity stage (Table 2). All these are used for classifying the folk species, varieties and sub-varieties. Most of the traits with the exception of seedling vigor (*Unkura gaarii*), leaf number (*Lakkofsa baala*), seed shattering (*Harca'u firii*) are used by over 90% of the farmers. These groups of traits are phenotypically vivid and are very commonly used by the farmers (Plate 7). Morphological traits such as panicle type (*Metansa*), seed colour (*Qlama shanyi*), threshability (*Callessu*), plant height (*Dhabata biqila*), stem sweetness (*Aalaa ta'ufii mi'aa*) are used by 100% of the farmers for classifying folk species. The less usage of seedling vigor, leaf number and seed shattering vis-à-vis other morphological traits might be due to less variation for these traits among folk species, varieties and sub-varieties. Comparison of the use of morphological related traits across altitudes showed that there is a significant variation for the proportion of usage for the leaf number (*Lakkofsa baala*), awns (*Qarma*), glume colour (*Qlama qolaa*), seed shattering (*Harca'u firii*), and maturity (*Bilchala*). This might be due to higher diversity for leaf number, awns and seed shattering in the highland and intermediate altitude and for glume colour in the lowlands. On the contrary, no significant variation was observed for the other morphological traits.

#### Biotic and abiotic stress related traits

According to the reaction to the various stresses, farmers classify their varieties (Table 2). In comparison to the morphologically-related traits, the groups of biotic and abiotic stress related traits are comparatively used less. There are two reasons for less usage of the traits: on-farm genetic variation is limited and stresses are not prevalent. To some of the stresses, such as frost, aphid, grain mold, ergot there are literally no performance variation for the folk species for other traits such as drought resistance (*Dheebuu danda'u*), lodging resistance (*Jigaw dhabu*), and low soil fertility tolerance (*Lafa dutuu danda'u*) farmers use them very frequently. However, significant variation for the proportion of usage for shootfly resistance, leaf disease resistance, head midge, lodging resistance, and drought resistance is observed. Stalkborer, *pokkah boeng*, leaf disease, and drought resistance are used more in the lowlands than in the highlands and intermediate altitudes. No variation in proportion of usage was observed for the other traits.

#### Use-related traits

As farmers over years have selected their varieties for multipurpose values, they do classify them according to the use values varieties render (Table 2). With the exception to stalk marketability (*Gurguraa qaraa*) (49.6%), milling quality (*Dakamufi mija'u*) (26.8%) and construction value (*Bu'a jaaru*) (74%), 90% of the farmers used other use related traits for folk classification. There is no significant variation for the proportion in the use related traits except for fuelwood value. The farmers in the highland and intermediate altitudes used sorghum stalk more as fuelwood compared to the lowlands. Hence, by the same token, the traits are used more in the same for classification.

### Folk classification tree in the highland, intermediate and lowland ecologies

Farmers classified their gene-pools by hierarchical classifications into parts that represented distinguishable groups of accessions beginning at the folk generic level and descending to the variety level (Table 3). The term *Mishinga *is used generally for all panicle-bearing sorghums. Actually in pearl millet growing areas such as Dire Dawa and Meisso, some of the farmers named pearl millet as the 'little sorghum' and thus they grouped into sorghum genera by virtue that pearl millet is panicle bearing. The first division distinguished sorghum from other cereals and named it as *Mishinga*, while the second division distinguished different sorghum types: wild (non edible) and cultivated (edible) sub-generic group (Table 4), and the third division is the folk species types namely *Muyra, Wegere, Fendisha, Chefere *(Figure 2, 3, 4) and the fourth division classifies into different varieties within the folk species.

In the course of diversity fairs when samples were collected for on station evaluation of diversity, farmers were mocked when I took samples from the non-edible sorghums '*Actually a farmer said to me that after all these 4–5 hours discussion this boy still does not understand what good sorghum is. He is collecting sorghum of donkeys, bird sown sorghums etc., actually, they were advising me that never take these samples as sorghum from our field so that they are not disgraced*'. Actually, this signified the strong use and functional differentiation criteria in folk taxonomy.

#### Farmers' sorghum classification tree

Based on the folk taxonomy descriptions, the folk sorghum classification trees were generated in lowland, intermediate and highland (Figure 2, 3, 4; Plate 8). It was learnt in this study for existence of variations and communalities in the folk species and varieties grown in the three ecologies. In the folk classification tree, there were some folk species, which were ubiquitous by name but they are ecologically differentiated and developed into ecotypes. This folk classification tree is based on three characters just as an example, but they were not exhaustive lists of folk species in each sorghum production environment.

From these folk taxonomy trees, it can be inferred that, with three characters (if we have two classes per character), a minimum of six varieties are expected. Hence, it is possible to expect that with more characters, more varieties per folk species are expected. Many of the folk species have varieties varying from two to eight. An example is shown by taking three folk species, *Wegere, Muyra *and *Fendisha *(Table 5).

### Folk names and their meaning

Farmers name their varieties in relation to introduction, use, use-related traits and morphological attributes and this is partly due to an 'implicit standardisation' of the folk taxonomic knowledge. The average farmer growing these varieties can name about 10–15 folk species (Plate 5, 6), a number which varies according to the indigenous technical knowledge, crop ecology and socio-economic conditions of the farmer. The average number of varieties encountered on farm is 9.07. The mean number of varieties, for the highland, intermediate and lowland areas amounted to 8.13, 10.95 and 8.12 respectively. It was found that the majority of the farmers use varietal mixture regardless of the crop growing ecologies.

#### Introduction

one of the major breeding methodologies used by the farmers is introduction. Following introduction farmers have been adopting varieties by their original names or modifying the name after the introducer of the variety into the area (Table 6). This is actually one of the recognition given by the adopting farmers through informal patenting either for the introducer or for the place of origin. Farmers gave names for the introduced varieties. It is normally given by the farmers who have received it for the first time.

#### Use and use related traits

one of the ways of naming folk species is to name it after the unique use it renders or use related traits it is endowed with (Table 7). This may refer indirectly to the chemical composition of the seed (e.g. *Daddu*), or stalk sweetness (e.g. *Alahserif*), dough property of the flour (e.g. *Bare Bekeyes*) or any other use associated part of the plant.

#### Morphological attributes

the different morphological attributes are also used by farmers to name the varieties (Table 8) (Plate 9, 10). Varieties are also named based on the morphological traits and related functions. For example: seed/grain related such as *Aday, Bullo, Firelemi, Keyla, Matsugi, Solle, Umrahajeria*; Glume related: *Firekolef, Kirmi*; Panicle related: *Anchero, Fechee, Merturasse, Nanno, Tomma, Tufkuur, Ilmijamma, Worabe*; Early maturity/drought resistance related: *Bele Melik, Chamme, Kumash, Meleta, Sheridon, Afukanni, Wourdi*; and plant height related: *Butene, Gebabe, Jilbeb*.

In sorghum folk taxonomy, some farmers were obviously casual and careless in naming panicles, while others were very careful and serious. This was noticed in pile sorting of sorghum panicles farmers brought in the diversity fairs. The latter were generally able to discuss the characteristics of a particular panicle and why it should not be classed as a particular variety. This indicates the presence of *de facto *rules and criteria in the naming and classification of panicles. Rules and criteria were also evidenced when groups of farmers were presented with different panicles to name. A name would be forwarded and argued in the course of focus group discussion until everyone arrives at consensus about the naming.

It may be assumed that the naming system of farmers is closely related to their knowledge of sorghum. Women and men have similar knowledge. Most commonly grown cultivars are constantly named while unpopular ones and varieties provided by government and non-government organizations are having various names in different localities.

In general, of the identified folk species, 16 were named after the introducer and/or geographical origin, 37 according to botanical traits, and 8 to use related traits. However, the meanings of some other folk species/variety names are not known by the farmers. This might be due to the discontinuity in knowledge transfer from some places to the other or the difference in the initial language used for naming. For instance, the origin for naming of the folk species such as *Gedineki, Fitibile*, and *Tomma *is not known.

### Comparative distribution and importance of folk species/varieties

The folk species importance and distribution varied from one place to another. Some of the folk species were dominant in some region but not in the other while there were some that were distributed all over the region. Some of the folk species might have been heard of by the farmers but could not be seen, have seen but not grown (Table 9). This shows that: first, there needs to be redistribution of the folk species into adapted zones so that other farmers can get considerable benefit; and second, the variation in adaptation from narrow to widely adapted types. Some of the sorghum types, like, *Wegere *(Plate 11), *Fendisha *(Plate 12), *and Muyra *(Plate 13) folk species have been found to grow in all regions, namely highland and intermediate and lowland ecologies while others are restricted to specific ecological niches.

**Table 9:** Proportion of folk species/varieties heard of, seen and grown by the farmers

Folk species/varieties	Heard	Seen	Grown	Folk species/varieties	Heard	Seen	Grown
*Aday*	2.0	0.4	1.2	*Kenya*	0.8	0.8	0.8
*Afukanni*	25.6	25.6	8.4	*Keremendie*	6.0	6.0	6.0
*Alasherif*	3.6	2.8	0.4	*Kereyu*	2.8	2.4	0.8
*Alemo(Ala-mo)*	0.0	0.0	0.0	*Keyla*	47.6	45.6	6.4
*Alegrad*	0.8	0.8	0.8	*Kirmi*	5.4	4.8	2.4
*Alisho*	1.6	4.4	2.4	*Kuffanzik*	3.2	2.4	2.0
*Ammajicta*	8.4	8.4	8.4	*Kuffe diffe*	25.6	18.0	0.8
*A(H)nchero*	11.2	10.4	3.6	*Kuffa kassa*	14.4	12.8	4.0
*Arebe (Umrahjeria)*	2.8	2.8	1.6	*Kumash*	1.6	1.6	0.0
*Assegid*	2.4	2.4	2.0	*Kuna*	4.0	2.8	1.6
*Bare Bekeyes*	3.8	3.6	1.6	*Manahaile*	4.0	3.6	2.4
*Bedukanni*	4.0	4.0	3.6	*Marur*	2.4	2.4	1.2
*Bele Melik*	30.8	23.6	2.0	*Masengo(Kelafo)*	9.6	7.2	1.2
*Beker*	1.6	1.6	1.6	*Matsugi*	43.6	39.6	10.4
*Bisidimo*	4.4	3.2	1.2	*Meko*	2.0	2.0	2.0
*Bullo*	15.2	13.2	1.2	*Meleta*	18.0	15.6	0.8
*Butene*	3.2	2.8	1.6	*Merturasse*	9.6	8.8	1.2
*Chamme*	12.4	12.0	6.0	*Merulae*	9.6	8.8	1.2
*Cherchero*	17.2	16.0	6.0	*Mullu*	3.6	2.4	1.2
*Chiquere*	60.8	60.4	6.0	*Mureta*	3.2	3.2	2.0
*Chomme*	14.8	12.8	1.2	*Muyra*	88.8	88.8	54.0
*Daddu*	2.0	1.6	0.8	*Nanno*	10.4	9.2	3.2
*Daar*	4.0	2.8	0.0	*Nolle*	7.2	6.4	0.8
*Danga*	32	28.4	3.6	*Okesedie*	2.4	2.4	0.8
*Dangasha*	6.0	6.0	6.0	*Rendie*	0.8	0.8	0.8
*Daslee*	40.8	37.6	12.8	*Serendie*	6.0	6.0	3.6
*Dengogof*	14.8	14.8	0.8	*Shefere*	6.8	6.8	5.2
*Dimma*	5.6	5.2	0.0	*Sheke*	4.8	4.8	4.8
*Dinni*	2.8	2.4	1.2	*Sheridon*	4.0	3.2	1.2
*Dulla*	6.0	5.2	0.4	*Shewe*	0.8	0.8	0.8
*Eja*	3.2	2.8	2.0	*Solle*	4.4	3.6	0.8
*Engidawork*	4.0	2.4	1.2	*Suta*	3.6	3.2	2.0
*Fechee*	15.6	13.6	2.8	*Wegere*	78.2	77.6	23.6
*Fendisha*	88	87.6	46	*Wobere*	2.0	2.0	0.8
*Firekolef*	0.8	0.8	0.0	*Wourdi*	5.6	4.4	1.6
*Firelemi*	4.6	3.0	2.4	*Taabelaa*	4.0	4.0	0.8
*Fitibile*	49.2	48.0	6.0	*Togge*	3.2	2.8	1.6
*Gebabe*	69.6	68.4	20.4	*Tomma*	5.6	4.8	0.0
*Gedi Neki*	14.8	13.2	1.2	*Tommis*	6.4	6.4	5.2
*Hado*	7.6	6.8	1.2	*Tuche*	2.4	1.6	5.2
*HajiiAli*	8.0	6.8	3.6	*Tufkuur or Bure*	14	10.4	0.4
*(H)Ifaato*	6.8	6.0	1.6	*T-76*	2.8	2.4	2.4
*Hamdye*	4.0	4.0	2.4	*Wahelo*	2.0	2.0	0.8
*Harkebasse*	8.8	8.8	3.6	*Worabe*	57.6	56.0	12.4
*Ilmijamma*	2.0	2.0	2.0	*Waybat*	6.0	5.2	0.8
*Jeldi*	36.0	34.8	11.2	*Wedayger*	2.4	1.6	0.8
*Jefere*	4.4	4.0	2.8	*Wedayjil*	0.4	0.4	0.4
*Jilbeb*	10.0	8.8	2.0	*Weliso*	5.2	5.2	1.2
*Jorro*	1.6	1.6	1.2	*Yade*	2.4	2.4	1.2
*Kassim*	3.6	2.0	0.8	*Yemen*	10.8	9.6	0.8

Except *Alamo*, most of the folk species have been heard of by the farmers in different occasions. The proportion of farmers who have heard of various folk species ranged from 0% (*Alamo*), 57.6% (*Worabe*), 60.8% (*Chiquere*), 69.6% (*Gebabe*), 78.2% (*Wegere*), 88% (*Fendisha*) to 88.8% (*Muyra*) and others are less than 50%. For *Alamo*, the reason could be that most of the lists of these folk species were developed in the course of participatory rural appraisals and on farm monitoring. However, in the course of individual interviews, it did not come out. This shows the implicit effect of the various survey methods employed.

The proportion of farmers who have seen folk species ranged from 0% (*Alamo*), 68.4% (*Gebabe*), 77.6% (*Wegere*), 87.6% (*Fendisha*) to 88.8% (*Muyra*). However, the proportion seen and heard of is equal for *Alamo, Alegrad, Ammajicta, Arebe, Assegid, Bedukanni, Beker, Dangasha, Kenya, Meko, Keremendie, Meko, Mureta *and *Muyra*.

The proportion of folk species grown ranged from 0% (*Alamo*), 20% (*Gebabe*), 23.6% (*Wegere*), 54%) (*Muyra*) to 64% (*Fendisha*). The following folk species namely, *Dangasha, Kenya, Keremendie, Meko, Rendie*, and *Sheke *have been equally heard of, seen and grown. While for the other folk species, the proportion of growers is less than those that have seen and much less than those that have heard of.

On the contrary, *Tomma, Daar, Dimma, Firekolef *and *Kumash *which used to be grown are no more grown currently. Hence, further investigation has to be made in order to conserve these folk species *ex situ*. These are high priority folk species for conservation but they are less important on farm and hence expensive to conserve *in situ*. These results indicate the importance of *ex situ *conservation given that *in situ *conservation may be an undue burden on the farmers.

### Folk taxonomy consistency

Folk taxonomy, like other indigenous technical knowledge, might have a problem of consistency. Hence, consistency of the naming system was the key issue for validation. In this study, sorghum naming system was not random. Based on the different classification criteria, farmers could distinguish most of the folk species. It was noticed during participatory rural appraisals and on farm monitoring that other member of the family, even children, could identify the different folk species and their naming. At least they know that they are different folk species, even if sometimes they might not know the actual folk species names.

**Figure 5:**
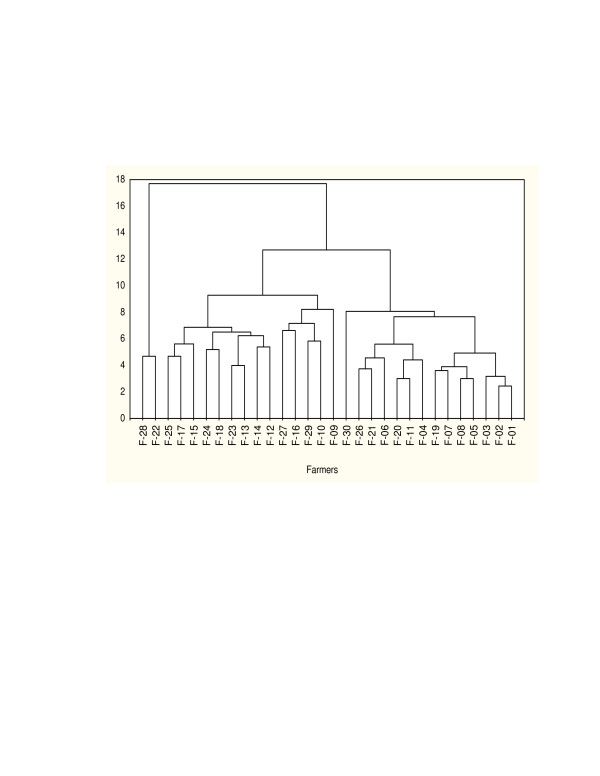
Clustering of farmers' consistency on folk taxonomy knowledge (F = Farmer; = Cluster).

The folk species/varieties used in the consistency study were collected from lowland parts of eastern Ethiopia including Babile where farmers were questioned for folk taxonomic consistency study. Of all the accessions planted in the field only 44 were subjected to the folk taxonomy consistency study. Farmers used various morphological, adaptive and use-related traits in identifying the folk taxonomic group. As to consistency, however, there were variations among farmers in folk taxonomic knowledge (Table 10). These variations were observed at three levels (1) Unknown folk species/varieties-this ranged from one to eleven. Farmers clearly indicated they do not know the folk species and varieties (2) Misnaming and misidentification-farmers misnamed and misidentified the varieties with what they know. This has happened because of the similarity of the tested varieties with what they grow both in seed and panicle types. This scenario was quite corroborated from the formal taxonomic perspective where compact to semi compact panicle types of Durra (D), Caudatum (C), and Durra-Caudatum (DC) races were prevalent in the lowland areas. In view of this, misnamed folk species/varieties ranged from 6 to 32. One thing that was evident during the evaluation was the presence of both 'lumpers' and 'splitters'. (3) Farmers skill was also different in the number of folk species/varieties identified. Only one farmer has identified 50% of the 44 folk species presented. Some are careful and good taxonomists others are careless in saying '*all is sorghum at least it is not maize'*. Properly identified species ranged from ten to twenty-three. Most of the farmers identified the dominant folk species and varieties grown around Babile. Based on the level of species not known, misnamed and identified properly the 30 farmers were grouped into four clusters (Figure 5).

**Table 10:** The proportion of farmers who have correctly named and identified folk species of the lowland and intermediate ecologies

Farmer (F)	Do not know		Not identified properly		Known properly	
	
	N	%	N	%	N	%
F1	3	6.8	28	63.6	13	29.5
F2	1	2.3	32	72.7	11	25.0
F3	6	13.6	28	63.6	10	22.7
F4	4	9.1	28	63.6	10	22.7
F5	3	6.8	27	61.4	14	31.8
F6	6	13.6	27	61.4	11	25.0
F7	4	9.1	26	59.1	14	31.8
F8	3	6.8	28	63.6	13	29.5
F9	7	15.9	24	54.5	12	27.3
F10	6	13.6	21	47.7	17	38.8
F11	3	6.8	29	65.9	12	27.3
F12	9	20.5	22	50.0	13	29.5
F13	11	25.0	12	27.3	21	47.7
F14	8	18.2	18	40.9	18	40.9
F15	11	25.0	16	36.4	17	38.6
F16	8	18.2	19	43.2	10	23
F17	7	15.9	6	13.6	23	52.3
F18	6	13.6	23	52.3	15	34.1
F19	5	11.4	27	61.4	12	27.3
F20	4	9.1	26	59.1	14	31.8
F21	8	18.2	24	54.5	12	27.3
F22	9	20.5	24	54.5	11	25.0
F23	6	13.6	26	59.1	12	27.3
F24	8	18.2	22	50.0	14	31.8
F25	9	20.5	18	40.9	17	38.6
F26	8	18.2	22	50.0	14	31.8
F27	9	20.5	22	50.0	13	29.5
F28	9	20.5	20	45.5	14	31.8
F29	4	9.1	25	56.8	14	31.8
F30	7	15.9	24	54.6	12	27.3

### Comparison of folk and formal taxonomy

Actually, a comparison of folk and formal taxonomy (Table 11) showed that there is certain degree of consistency with tolerable variation. Besides, race is not a scientific unit to be used in classification though it has recognizable morphological identity with some genetic integrity. As a biological unit, race is not clearly separable as species but has a distinct cohesion of morphology, geographical distribution, ecological adaptation and frequently breeding behaviour. However, the racial differentiation is not always clear, there are ill-defined races, hybrid races and races in the process of formations. It must be emphasized that races and sub-races are not intended to be formal categories and they are not to be italicised. Hence, the inconsistency observed for folk taxonomy when cross checked with formal taxonomy is not unexpected.

**Table 11:** A comparison of folk and formal taxonomy

Folk species	Harlan and de Wet race classification (1972)	Folk species	Harlan and de Wet race classification (1972)
*Abdelota*	*D*	*Firekolef*	*DC*
*Afukanni*	*DC, D*	*Gebabe*	*DC*
*Ammajicta*	*D, DC, C*	*Gedineki*	*D*
*Arebe*	*D, DC*	*Goronjjo*	*D*
*Bele Melik*	*DC*	*Ilmijamma*	*DC, D*
*Bullo*	*D, C*	*Jorro*	DC
*Chamme*	*DC, D*	*Jefere*	*DC*
*Cherchero*	*DC, D*	*Jeldi*	*D, DC*
*Chefere*	*DC*	*Kirmi*	*DC*
*Chiquere*	*DC*	*Limai*	*GC*
*Chomme*	*D*	*Merturasse*	*DC*
*Danga*	*DC*	*Murata*	*DC, D*
*Dimma*	*D*	*Muyra*	*DC, D*
*Danga*	*DC*	*Mesengo*	*D, C*
*Dinni*	*DC*	*Nanno*	*DC, B*
*Fendisha*	*DC*	*Taabela*	*D*
*Firelemi*	*DC*	*Wahilo*	*D*
*Harchatee*	*DC*	*Wegere*	*DC, D*
*Fechee*	*DC*	*Worabe*	*DC*
*Fitibile*	*DC*	*Wourdi*	*DC*
*Fechatee*	*DC*	*Zengada*	*DC*

Legend:*D *= Durra; *C *= Caudatum; *B *= Bicolor; *DC *= Durra-Caudatum. Sorghum classification according to Harlan and deWet race classification ***Crop Science***. (1972). 12:172–176

Folk species such as *Ammajicta*, *Afukanni*, *Chamme*, *Ilmijamma*, *Jeldi*, *Murata*, *Muyra*, *Mesengo*, *Nanno*, and *Wegere*, have more than one race. However, for the other folk species there was one-to-one correspondence to the races. The inconsistency from formal taxonomy can be seen, if reversed: for instance, Durra-Caudatum (DC) race includes 32 of the 42 listed folk species. Hence, what has been split by folk taxonomy has been lumped by formal taxonomy and vice versa.

## Discussion

Harlan and de Wet [18] genetically classified sorghum into primary gene pool, secondary gene pool, and tertiary gene pool; this was also partly encountered in folk taxonomy. For instance, some farmers are trying to classify pearl millet into sorghum, which is a secondary gene pool for sorghum. Farmers classified sorghum into cultivated and wild species (spontaneous races); this is compatible with primary gene pool of sorghum [19]. Genetically, edible sorghum and non-edible ones belong to the same biological species.

The classification of wild and cultivated sorghum has been accommodated by dynamic property, non-exclusive and flexibility in classification of folk taxonomy. As pointed out by Berlin *et al*., [20], these are common characteristics of folk biology. In the course of classification and naming, the following scenarios particular to folk taxonomy have been encountered which implied for the use of integrated folk-formal taxonomy.

### Polysemics

the same name can refer to different varieties. *Afukanni *and *Kuffa Kassa *in the intermediate and lowland areas refer to different varieties, though the different varieties have the same name. For example, *Afukanni *refers to an early maturing variety i.e. varieties with other different characteristics but which matures early is called *Afukanni*. It is more of a general name referring to early maturing varieties. Polysemics was also encountered in improved varieties. A drought resistant variety either improved or farmers can be named as *Kuffakassa *because of drought resistance *per se*. This is a lumped name. However, improved variety T76 #23 is called *Afukanni *in Babile and in Dire Dawa, indicating the consistency in polysemics in different localities.

### Semantics

*Shefere, Jefere, Chefere*; *She*', *Je*' and *Che*' are the semantics used in different localities, they might represent the same folk species. *Fechee *and *Fechatee *might refer to the same folk species but the way it is called varied in the different locality.

### Multiple names for one folk species/variety

One variety can have more than one name: *Zengada *(by *Amhara *farmers), *Fitibile *and *Chiquere *(by *Oromo *farmers); *Arebe *(coming from Arab countries), *Yemeni *(possible origin of the variety might be from Yemen) and *Umrahjeria *refer to the same variety but named after its whitish rocky seed colour.

### New names are given to some varieties within one folk species

For instance, one of the varieties of *Muyra *folk species is named as *Hamdye*, as it is an earlier maturing and drought resistant type. *Keyla *is one type of *Fendisha *folk variety that has light red seed colour and insipid stalk types.

### Naming of Improved Varieties

Few varieties which are adopted by the farmers in the lowland areas are given various names depending on the conditions. For example, an improved variety-*Gambella *1107, in Dire Dawa, is named as *Kuffa Kassa *(because of its drought resistance), *Afukanni *(because of its early maturity), *Manahile *(introduced by farmer *Manahile*), *Esapako *(its time of introduction coincides with Socialist Party System). *Afukanni *is given to *Gambella *1107, T76#23 and IS 9302 because all of them do mature earlier than farmers varieties.

The folk names are sometimes limited by the relative importance of folk species and varieties to particular village (*ganda*), farming community, ethnic groups or *weredas*. As indicated in Table 9, the importance of folk species varied and most of them have increased local importance with the exception to some folk species that were widely and popularly known across the region.

### Unexplained names

The meaning of the names of some of identified folk species is not known. It is difficult to know unless the people who named it or the place of origin is traced back. The original name is adopted and maintained with variety in the course of farmer-to-farmer dissemination. A similar pattern was observed in rice [12].

Folk taxonomic nomenclature is an integral part of the variety management in many farming systems [21-23]. In view of this, the multitude of names at various folk taxonomic levels indicated the prevalence of on farm genetic diversity at infra-specific level. This is also in agreement where folk taxonomy is used to highlight the amount of genetic diversity [24,25]. In this study, over 78 folk species (Table 9) have been identified which indicated the level of on farm genetic diversity. The variation among naming connotes geographical, genetical and ecological diversity. Diversity is reflected in the multiplicity of names farmers have been using for different folk species. This is also in agreement with ethno-biologists [26-29] who pointed out that rich folk knowledge is one of the factors accounting for maize diversity in Mexico.

It has been repeatedly shown that inter-specific folk taxonomy are accurate [26,30] but a doubt shadows over accuracy of intra-specific level. Formal taxonomy has failed most conspicuously at the intra-specific level in cultivated plants [9]. This has to be evident in that the formal taxonomy is using strict taxonomical parameters while the folk taxonomy, in addition, has functional, adaptive and use related parameters, which might result either in splitting or lumping of the class of formal taxonomy. This partly disagreed with the findings of Teshome et al. [18] in which there was full consistency.

Inconsistencies are not only within folk taxonomy but also in formal taxonomy [21]. Confusion in folk taxonomy extends over the generic, specific and infra-specific level. For instance in formal taxonomy of sorghum, Snowden [31] used 31 species of cultivated groups; Jackushevsky [32] reduced these to nine and de Wet and Huckabay [33] to one. The similarities in the folk species lead to confusion among the farmers and this was also encountered by Boster [34] which finally resulted in 'splitters' and 'lumpers' (Figure 5, Table 10). Similarly, Harlan [9] indicated that conventional formal taxonomy tends to over classify and provide too many categories. Hence, an informal system based on gene pools, races and sub-races is proposed.

Most of the folk taxonomy descriptors are in agreement with formal taxonomy ones developed by IBPGR/ICRISAT [35]. In addition new folk taxonomy descriptors related with glumes, panicles and nodal tillers have been identified to be used in the formal taxonomy.

The outer glume colours of sorghum as per ICRISAT/IBPGR descriptors are scored as a single colour. However, it was found that in the study area most of the glume colours compose more than one colour. Hence, the formal glume colour descriptors have to add categories of multi-colored glumes, e.g., *Jeldi *is partly straw-red glume coloured. Though theoretically there is one glume colour per head, the number of folk species with more than one colour per panicle was not few in this study. It can be uni-or multi-colored, e.g., *Fechatee*-straw and black coloured glumes per panicle. In the ICRISAT/IBPGR descriptors, it has never taken into account the inside glume colour. However, farmers clearly showed that varieties with similar outer glume colour but different inside glume colour, e.g, *Arebe *folk species.

ICRISAT/IBPGR descriptor for panicle types provide 12 type of panicles, which was comparable to the folk panicle descriptors. However, if we take the compact panicle type: it has only two versions, elliptic and oval in the formal one. On the contrary, farmers indicated at least four types of tips that are stable inherited by folk species and used as one of the distinct character for classifications. As indicated in Table 2, tip of the panicle can be flat, with tits, pointed or pigmouthed type. Hence, more categories of panicle types have to be included. Besides, the less accommodating formal descriptor categories for panicle types have been felt in the morphological characterisation of folk species such as *Muyra*, *Wegere*, and *Fendisha*.

In the ICRISAT/IBPGR descriptor, the normal evaluation of nodal tillers is for either absence or presence. Flowering synchrony is evaluated for basal tillers only. However, farmers indicated that flowering synchrony of nodal tillers is one criterion for folk taxonomy. Some of the folk species, for example *Bedukanni *folk species, have nodal tillers that synchronise in both flowering and maturity with the main tiller.

One of the salient importance's of folk taxonomy is that it makes genetic resources collection and conservation simple, practical and very objective. Actually, before any genetic collection, conservation, characterisation and evaluation, prior information and study on folk taxonomy is indispensable for systematic comprehensive germplasm collection. A lot of sorghum germplasm, closer to 8000, have been collected from Ethiopia [36]. This collection was made without having *apriori *information on folk taxonomy, which has resulted in the incomplete coverage of the collection by folk species. One of the reasons for the duplication or lack of exhaustive *ex situ *collection was due to lack of information on folk taxonomy in the course of collection and characterisation [37]. This completely agrees with the idea of Boster [38] that folk taxonomy of great intra-specific variability can be understood as a means to maintain diversity and that diversity is sought for its own sake and not for specific reason. These types of appropriate folk taxonomic studies have to be made in the centre of diversity and origin for enhancing genetic resources before the folk taxonomic knowledge is eroded.

Folk taxonomy can help in identifying relative value of folk species/varieties (Table 7 and 8) for proper characterisation and pre-breeding activities. A similar study on rice in Nepal has shown that name of the varieties indirectly related showed the functional value of the variety [12]. One of the major problems in breeding crops in centre of origin and diversity is the problem of improved varieties to perform better than farmer varieties mixture grown, as the farmers have many varieties in their hands [14-17]. Hence, for cultivars development in these areas, folk taxonomic classifications identify the important traits and varieties enhancement program, crossing and evaluation. Besides, complementary varietal components for varietal mixture can be bred.

Knowing folk taxonomy helps to identify the importance and distribution of the folk species and hence helps to develop an *in situ *conservation scheme for farmers' varieties. As indicated in Table 9, the relative importance of folk species varied hence it can help to assess and prioritise folk species and varieties for *in situ *and *ex situ *conservation [37].

Sorghum was commonly exchanged and distributed according to the folk names [16,17]. Exchange of sorghum between families was commonly referred to in terms of specifically locally named varieties. Farmers in one village generally know which households certainly have named varieties and their particular agronomic and use value-related characteristics. When asked about a particular variety, farmers often reported that they did not have it but that it could be found in a particular household and where it was cultivated. Knowing folk taxonomy also helps in developing seed distribution, flow networks and regional varietal map.

## Conclusion

Folk taxonomy, like that of most indigenous technical knowledge, has some drawbacks. This is due to the fact that 'the standards in folk taxonomy' are not expected to be absolutely consistent across all ecological regions, social and economic domains, there are some anomalies. Similarly, formal taxonomy has its weaknesses. In view of the strength and weakness of folk and formal taxonomy, integrated folk-formal taxonomy will be imperative for management and utilization of on farm genetic resources.

The benefits that are implicit from folk names are the reflection of associated values of each folk species. Farmers over years have identified the nutritional, medicinal and other values of varieties that will definitely help for selection of varieties for directed plant breeding.

Folk taxonomy names are sometimes limited due to distribution, importance, and adaptation:

1. There is local adaptation of folk species through their ecotypic forms.

2. Farmers specialize in some folk species.

3. Farmer-to-farmer dissemination of varieties happens at very slow rate.

The most important morphological trait used in folk taxonomy is panicle-related traits, which is similar with the formal taxonomy standards developed by Harlan and de Wet [18]. Hence, characterisation, selection, and crossing based on panicle-related traits will have a definite role for on-farm plant genetic resource management and breeding.

In order to use the developed folk species and consolidate folk taxonomy it would be essential that the existing farmers' classification be complemented with morphological, biochemical and molecular markers study.
